# Recurrent *Candida albicans* Ventriculitis Treated with Intraventricular Liposomal Amphotericin B

**DOI:** 10.1155/2015/340725

**Published:** 2015-10-08

**Authors:** Demet Toprak, Sevliya Öcal Demir, Eda Kepenekli Kadayifci, Özden Türel, Ahmet Soysal, Mustafa Bakir

**Affiliations:** Department of Pediatrics, Marmara University School of Medicine, 3500 Istanbul, Turkey

## Abstract

Central nervous system (CNS) infection with *Candida* is rare but significant because of its high morbidity and mortality. When present, it is commonly seen among immunocompromised and hospitalized patients. Herein, we describe a case of a four-year-old boy with acute lymphoblastic leukemia (ALL) who experienced recurrent *Candida albicans* meningitis. The patient was treated successfully with intravenous liposomal amphotericin B at first attack, but 25 days after discharge he was readmitted to hospital with symptoms of meningitis. *Candida albicans* was grown in CFS culture again and cranial magnetic resonance imaging (MRI) showed ventriculitis. We administered liposomal amphotericin B both intravenously and intraventricularly and favorable result was achieved without any adverse effects. Intraventricular amphotericin B may be considered for the treatment of recurrent CNS *Candida* infections in addition to intravenous administration.

## 1. Introduction

*Candida* infection of central nervous system (CNS) is relatively unusual in children. It is commonly reported among premature neonates and immunocompromised or hospitalized patients. Various studies reported its mortality to be between 10% and 33%, with a morbidity of about 14% [1, 2]. Debates continue regarding the most appropriate treatment for* Candida* meningitis and ventriculitis. Current guidelines suggest that the first choice is either single intravenous amphotericin B or a combination of intravenous amphotericin B with flucytosine (5-FC), but these options may not be sufficient for cure [3, 4]. Few cases with* Candida* CNS infection were previously reported to be treated with intravenous amphotericin B which is generally believed to have toxic effects on the neural tissue [5–8].

Herein, we present a four-year-old child with recurrent* Candida albicans* ventriculitis treated with intraventricular liposomal amphotericin B in conjunction with intravenous liposomal amphotericin B.

## 2. Case Report

A four-year-old boy with acute lymphoblastic leukemia (ALL-L2) was admitted to the hospital with diagnosis of febrile neutropenia with an absolute neutrophil count of zero. He was at the end of remission induction phase of ALL-BFM 2003 protocol. Neck stiffness and acute mental status changes were observed on his physical examination. His cranial MRI showed generalized hydrocephalus; lumbar puncture was performed and analysis of the cerebrospinal fluid (CSF) specimen revealed 312 white blood cells/*μ*L, 23 mg/dL of glucose, and 126 mg/dL of protein. Vancomycin and meropenem were initiated empirically with the diagnosis of meningitis. Repeated lumbar puncture performed since his symptoms persisted despite the five days of antibacterial treatment; CSF analysis revealed 160 white cells/*μ*L, 53 mg/dL of glucose, and 160 mg/dL of protein.* Candida albicans* grew in CSF culture in 2 days; therefore, intravenous liposomal amphotericin B with a dose of 5 mg/kg/day was added to the antibacterial treatment regimen. Because the signs and symptoms of hydrocephalus continued to progress, an external ventricular drainage device was inserted. During the follow-up, he showed significant clinical improvement in addition to improved CSF profile. Liposomal amphotericin B was discontinued at the 30th day of treatment, because CSF cell count and glucose and protein levels returned to normal levels and the last three CSF cultures kept sterile. The drainage device was replaced by a ventriculoperitoneal shunt and the patient was discharged.

Twenty five days after discharge, he was readmitted to the hospital with signs and symptoms of hydrocephalus. Cranial MRI revealed generalized hydrocephalus and ventriculitis.* Candida albicans* grew in the CSF culture again. Liposomal amphotericin B was given both intravenously (5 mg/kg/day once daily) and intraventricularly (1 mg/day, dissolved in 3 mL of 5% dextrose, and shunt was closed for 4 hours after 4 administration). The patient also underwent extraventricular drainage to treat the hydrocephalus. Neurological or nonspecific side effects like vomiting, headache, or paralysis were not observed during intraventricular amphotericin B treatment. Intraventricular liposomal amphotericin B treatment was discontinued at the 12th day because the sterilization of CSF was achieved in addition to normalization of CSF WBCs, glucose, and protein levels. Intravenous liposomal amphotericin B treatment was continued for six weeks. Ventricular drainage device was replaced by a new ventriculoperitoneal shunt before discharge.

## 3. Discussion

Treatment guidelines for* Candida* meningitis are still not well defined. However, a lipid formulation of amphotericin B given intravenously (3 to 5 mg/kg once daily), either with or without flucytosine, is recommended [3, 4]. We used liposomal amphotericin B alone in our case because flucytosine is unavailable in our country.

Amphotericin B is the drug of choice because of its fungicidal activity against almost all* Candida* species [9]. Various treatment outcomes have been observed with other antifungals. Fluconazole is highly effective against most* Candida *spp. and has a very good CSF penetration. However, therapeutic failures with this drug have been reported [10]. An animal study suggests that amphotericin B sterilizes the CSF faster than fluconazole [11]. Oral fluconazole after an initial course of liposomal amphotericin B was recommended if the* Candida* spp. is susceptible [4, 12]. The clinical experience with voriconazole is limited for* Candida* CNS infections, and additionally posaconazole, caspofungin, and other echinocandins do not achieve adequate CSF concentrations to treat* Candida* meningitis [4, 13, 14]. Thus, until the evidences of their efficacy are demonstrated, they should not be used in treatment of CNS candidiasis. Due to the absence of antifungal susceptibility test in our laboratory, we continued the treatment with amphotericin B.

The brain and CSF concentration of amphotericin B remains low during its intravenous administration [9]. There are few case reports about intrathecal or intraventricular administration of amphotericin B in the literature [5–8]. Because of its toxic effects on the CNS such as chemical arachnoiditis, ventriculitis, acute encephalopathy, and myeloradiculitis [10], such an approach can be used for patients in critical condition or for those who have been intolerant to systemic amphotericin B. Udeta Mora and Sanches Perez [5] first reported in 1979 a premature infant with* Candida* meningitis who was treated with amphotericin B intravenously for 41 days and intraventricularly for 21 days. During the treatment, the patient was reported to develop purulent meningitis. In 1983, Galli et al. [6] reported a seven-year-old girl with inoperable disseminated cerebral aspergillosis treated with amphotericin B both intravenously and intraventricularly. Intraventricular administration of amphotericin B was well tolerated and did not cause any adverse effects. In the same year, Fisher and Dewald [7] reported a young woman with cryptococcal meningitis treated with intraventricular amphotericin B who developed transient signs of parkinsonism during treatment. In 1989, Shapiro et al. [8] reported seven cases of CNS shunt infections with* Candida albicans*. All patients were successfully treated via shunt removal and intravenous amphotericin B, but four of them also received intraventricular amphotericin B as part of their treatment.

Recurrence of* Candida* meningitis in our case about a month after intravenous amphotericin B treatment suggests a treatment failure due to residual yeast in ventricles. Rather than taking a risk of recurrences, we decided to add intraventricular amphotericin B in addition to intravenous route, since the 5-FC was unavailable in the country. During the 6 weeks of treatment no neurological or other side effects were observed.

There are no widely accepted guidelines with regard to the duration of antifungal treatment for CNS fungal infections. Antifungal chemotherapy is usually recommended until an MRI shows that all abscesses, if present at presentation, have disappeared, the CSF WBC count, glucose, and protein levels have returned to normal, culture remains sterile, and the patient's symptoms and signs have resolved [3]. However, it may take weeks or even months for all of the above to be accomplished. Repeating the lumbar puncture is suggested after one to two weeks of therapy to ascertain whether the WBC count is decreasing and CSF is sterilized. In our patient, after 10 days of treatment, the repeated lumbar puncture showed normal CSF glucose, protein, and WBC count levels, and sterilization of the CSF was achieved. Therefore, we stopped the intraventricular amphotericin B on the 12th day of treatment. During his follow-up for more than one year, he remained well without neurological symptoms.

As a conclusion, we believe that recurrent or persistent* Candida* ventriculitis may be successfully treated by administration of intraventricular and intravenous liposomal amphotericin B simultaneously.
